# Rapid microevolution during recent range expansion to harsh environments

**DOI:** 10.1186/s12862-018-1311-1

**Published:** 2018-12-07

**Authors:** Yiyong Chen, Noa Shenkar, Ping Ni, Yaping Lin, Shiguo Li, Aibin Zhan

**Affiliations:** 1 Research Center for Eco-Environmental Sciences, Chinese Academy of Sciences, 18 Shuangqing Road, Haidian District, Beijing, 100085 China; 2 University of Chinese Academy of Sciences, 19A Yuquan Road, Shijingshan District, Beijing, 100049 China; 3 School of Zoology, George S. Wise Faculty of Life Sciences, Tel-Aviv University, 6997801 Tel-Aviv, Israel; 4 The Steinhardt Museum of Natural History, Israel National Center for Biodiversity Studies, Tel Aviv University, Tel-Aviv, Israel; 5 GEOMAR Helmholtz-Zentrum für Ozeanforschung Kiel, Düsternbrooker Weg 20, 24105 Kiel, Germany

**Keywords:** Biological invasion, Rapid microevolution, Range expansion, Invasive species, Red Sea, Adaptive genes, *Ciona robusta*

## Abstract

**Background:**

Adaptive evolution is one of the crucial mechanisms for organisms to survive and thrive in new environments. Recent studies suggest that adaptive evolution could rapidly occur in species to respond to novel environments or environmental challenges during range expansion. However, for environmental adaptation, many studies successfully detected phenotypic features associated with local environments, but did not provide ample genetic evidence on microevolutionary dynamics. It is therefore crucial to thoroughly investigate the genetic basis of rapid microevolution in response to environmental changes, in particular on what genes and associated variation are responsible for environmental challenges. Here, we genotyped genome-wide gene-associated microsatellites to detect genetic signatures of rapid microevolution of a marine tunicate invader, *Ciona robusta*, during recent range expansion to the harsh environment in the Red Sea.

**Results:**

The Red Sea population was significantly differentiated from the other global populations. The genome-wide scan, as well as multiple analytical methods, successfully identified a set of adaptive genes. Interestingly, the allele frequency largely varied at several adaptive loci in the Red Sea population, and we found significant correlations between allele frequency and local environmental factors at these adaptive loci. Furthermore, a set of genes were annotated to get involved in local temperature and salinity adaptation, and the identified adaptive genes may largely contribute to the invasion success to harsh environments.

**Conclusions:**

All the evidence obtained in this study clearly showed that environment-driven selection had left detectable signatures in the genome of *Ciona robusta* within a few generations. Such a rapid microevolutionary process is largely responsible for the harsh environmental adaptation and therefore contributes to invasion success in different aquatic ecosystems with largely varied environmental factors.

**Electronic supplementary material:**

The online version of this article (10.1186/s12862-018-1311-1) contains supplementary material, which is available to authorized users.

## Background

Dissecting evolutionary dynamics of environmental adaptation in organisms is one of the fundamental research themes in evolutionary biology and ecology, particularly in the context of rapid global climate change and distributional shift of organisms in the past five decades [1–3]. Species spreading to and subsequently successfully colonizing new regions are, or will be, inevitably suffering from environmental changes/pressures [4, 5]. Adaptive evolution is one of the crucial mechanisms to facilitate species to survive and thrive in novel environments [6–8]. Recent studies suggest that adaptive evolution could rapidly occur in species (i.e., rapid microevolution) to respond to novel environments or environmental challenges during range expansion [9–12].

Rapid microevolution is usually associated with changes of genetic variation in a population, such as changes of allele frequency and generation of new alleles through mutations at functional genes [7, 13, 14]. Multiple studies clearly showed that evolutionary changes could occur within dozens of generations, as those studies detected significant evolutionary signatures using genetic markers in populations living in varied environments [15–17]. Available evidence suggests that such a rapid microevolutionary process could be driven by multiple factors and their complex interactions, such as selection (natural or artificial), gene flow, genetic drift and mutations [15, 16, 18, 19]. For example, rapid microevolution occurred in three-spine sticklebacks within less than 50 generations during habitat transitions from marine to freshwater habitats, and many important functional loci and genes associated with adaptive traits were involved in the process of rapid environmental adaptation [20, 21]. However, for environmental adaptation, most studies successfully detected phenotypic features associated with local environments, such as changes of morphological characteristics and life history traits, but did not provide ample genetic evidence on microevolutionary dynamics [15, 22]. Thus, it is crucial to deeply investigate the genetic basis of rapid microevolution in response to environmental adaptation, in particular on what genes and associated variation are responsible for environmental changes/challenges during range expansion [23–25].

Biological invasions are promising ‘natural’ experiments that provide opportunities to study rapid microevolutionary processes [10, 26, 27]. Invasive species inevitably face extremely rapid, or even sudden, environmental changes during range expansion, thus providing good materials to deeply investigate how species rapidly adapt to novel environments [26, 28, 29]. The highly invasive ascidian, *Ciona robusta*, is a notoriously fouling organism with wide salinity tolerance ranging from 12‰ to 40‰ and temperature varying from 3 °C to 30 °C [30, 31]. *C. robusta* is usually considered to be native to Northwest Pacific and has invaded the coasts of Mediterranean Sea, Atlantic, Pacific and Oceania oceans [31–33]. Most invasion events of this species have been recorded in temperate and sub-tropical waters, though a few populations appeared in tropical harbours with transitory states [34, 35]. In different environments, the life cycle characteristics of *C. robusta*, such as the growth rate, life span and spawning time, vary depending on local environmental factors, particularly the water temperature and salinity [30]. For example, populations in cold and temperate coastal regions could reproduce 2–3 generations per year [30, 36]; however, more than 4 generations/year were observed in warm waters, such as those in warm regions of the Mediterranean Sea [30].

A recent introduction of *C. robusta* has been reported in the Red Sea, a representative tropical region which is well known for its high temperature and salinity [35]. The first occurrence of *C. robusta* was detected in the Eilat marina in 2015 [35]. Many environmental factors in the Red Sea, such as water temperature (> 27 °C) and salinity (> 40‰, Table 1), represent the extreme in the distribution ranges of *C. robusta* reported so far. Genetic analyses based on mitochondrial DNA suggested that the Red Sea population might be introduced from the Mediterranean Sea through the Suez Canal [35]. Since the opening of the Suez Canal in 1869, a large number of non-native species have been introduced from the Red Sea to the Mediterranean Sea [37, 38]. For example, more than 90 fishes have been introduced through the Lessepsian migration, a process of biological invasions from the Red Sea into the Mediterranean Sea [37–39]. However, only a handful of opposite-direction introductions from the Mediterranean to the Red Sea (anti-Lessepsian migration) have been recorded so far [40], mainly owing to harsh environmental conditions in the Red Sea, such as high salinity, high temperature and oligotrophic conditions [41, 42]. For the Mediterranean *C. robusta* populations, the optimal temperature range was 14–23.4 °C [43]; however, the Red Sea population could survive and reproduce in such harsh environmental conditions, indicating a wider environmental tolerance than previously assumed. Thus, the recent successful range expansion provides good materials to study genetic signatures of rapid microevolution, particularly on genetic mechanisms of rapid local adaptation to severe environmental conditions in such a short timescale in the wild.

**Table 1:** Sampling sites and genetic diversity indices based on genome-wide gene-associated microsatellites of *Ciona robusta*

Site code	Region, Country, Ocean	Latitude	Longitude	AveT	MaxT	MinT	AveS	MaxS	MinS	*N*	*A*_R_	*H*o	*H*_E_	*F*_IS_
RS	Eilat, Israel, the Red Sea	29°33′11″N	34°57′35″E	24.97	28.67	22.04	40.34	41.35	39.72	22	2.524	0.349	0.427	0.186
AM	Arenys de Mar, Spain, the Mediterranean	41°33′41″N	2°34′37″E	17.98	25.28	13.29	37.67	38.21	36.87	48	2.853	0.307	0.416	0.264
BL	Blanes, Spain, the Mediterranean	41°41′12″N	2°53′22″E	17.44	24.38	12.98	37.95	38.32	37.48	22	2.624	0.307	0.442	0.311
SA	Cape Town, South Africa, the Atlantic	33°54′33″S	18°25′59″E	16.03	16.92	15.16	35.18	35.3	34.99	33	2.930	0.325	0.435	0.256
NMF	Nelson, New Zealand, the Pacific	41°15′29″S	173°16′42″E	13.55	16.37	11.22	34.78	34.92	34.62	17	3.937	0.399	0.562	0.296
GAP	Gampo, Korea, the Pacific	35°48′26″N	129°30′13″E	17.72	24.31	12.18	33.72	34.48	32.17	30	3.789	0.389	0.528	0.266

A total of six populations, including the population collected from the Red Sea in this study and other five populations from our previous study [44]. AveT, annual average water temperature; MaxT, the highest monthly average water temperature; MinT, the lowest monthly average water temperature; AveS, annual average water salinity; MaxS, the highest monthly average water salinity; MinS, the lowest monthly average water salinity; *N*, number of individuals; *A*_R_, mean allelic richness; *H*_O_, mean observed heterozygosity; *H*_E_, mean expected heterozygosity; *F*_IS_, mean inbreeding coefficient

In this study, we sampled the recently established population of *C. robusta* in the Red Sea and genotyped genome-wide gene-associated microsatellites to reveal the genetic signatures of rapid microevolution in the harsh environment. In order to cover various temperature and salinity gradients globally, we included five other populations (two Mediterranean populations, one Atlantic population, two Pacific populations) from Lin et al. [44]. At the genome level, we aimed to (i) investigate the population genetic patterns potentially shaped by local environmental conditions in the Red Sea, and (ii) detect adaptive genetic variation and genes involved in rapid environmental adaptation in the process of range expansion to harsh environments.

## Results

### Significant difference of environmental factors

Based on the gradients of sea water temperature and salinity (Table 1) and our previous population genetic studies [32, 33, 44], five other global populations from Lin et al. [44] were divided into three groups. Two populations (AM and BL) from the Mediterranean Sea were assigned into one group, the population SA from the Atlantic Ocean formed another group, and the remaining two populations (NMF and GAP) from the Pacific Ocean were the third group. Non-parametric Kruskal-Wallis test of six environmental factors showed statistical difference between the Red Sea population and other three groups (*p* < 0.01), suggesting significantly different environmental conditions in the Red Sea. The Red Sea population had significantly higher temperature and salinity than the other populations (Additional file 1: Figure S1, S2).

### Population genetic diversity and structure

A total of 22 individuals from the Red Sea were confirmed as *C. robusta* by molecular identification. Based on 146 genome-wide gene-associated microsatellite loci, we identified 975 alleles across all 22 individuals. Microsatellite diversity of the Red Sea population (i.e. *A*_R_ = 2.524) was significantly lower than that in the other five global populations (Kruskal-Wallis test, *p* < 0.01), especially two Pacific populations (NMF, *A*_R_ = 3.937; GAP, *A*_R_ = 3.789; Table 1). However, we did not detect the signal of recent population bottleneck with the TPM model, and the allele frequency of the Red Sea population exhibited a normal L-shape.

**Fig. 1:**
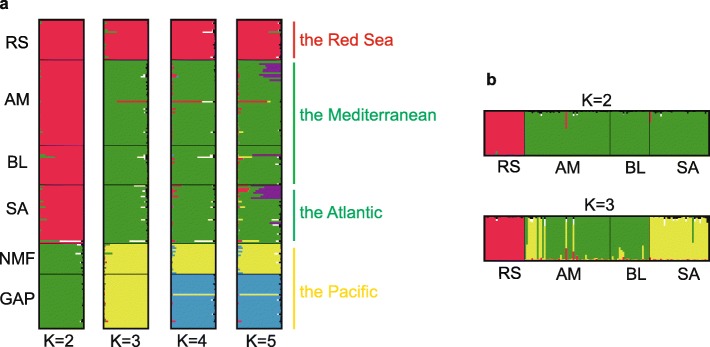
Bayesian inference population genetic structure of *Ciona robusta* in STRUCTURE. **a** *K* values from 2 to 5 based on the Red Sea population and other five populations; **b** *K* values from 2 to 3 based on four populations from the Red Sea, the Mediterranean sea and the Atlantic ocean, respectively. Each genotype is represented by a thin line, with proportional membership in different clusters indicated by different colors. Bold vertical lines separate collection sites, with sites ID as per Table 1

Significant genetic differentiation was detected between the Red Sea population and the others, with the pairwise *F*_ST_ values ranging from 0.053 to 0.215 (Table 2). The pairwise *F*_ST_ values were relatively low between the Red Sea population and two Mediterranean populations (AM, BL). In comparison, the highest genetic differentiation was found between the Red Sea population and one Pacific population collected from New Zealand (NMF, *F*_ST_ = 0.215; Table 2). The Bayesian assignment analyses in STRUCTURE based on all microsatellite loci suggested a two-cluster model (optimal *K* = 2). Individuals from the Red Sea population were assigned into the same cluster formed by three other populations (two Mediterranean populations: AM, BL; one Atlantic population: SA), whereas two Pacific populations (NMF, GAP; Fig. 1a) were grouped into the other cluster. However, the Red Sea population was clearly separated from the cluster of Mediterranean-Atlantic populations at higher *K* values (e.g., *K* = 3, 4, 5; Fig. 1a). Furthermore, the results confirmed that the Red Sea population exhibited genetically different structure when the Red Sea and Mediterranean-Atlantic group was re-analyzed separately (Fig. 1b).

**Table 2:** Population genetic differentiation (pairwise *F*_ST_) based on genome-wide gene-associated microsatellites of *Ciona robusta*

Population	RS	AM	BL	SA	NMF	GAP
RS	****					
AM	**0.056**	****				
BL	**0.053**	0.000	****			
SA	**0.068**	**0.017**	**0.029**	****		
NMF	**0.215**	**0.205**	**0.182**	**0.206**	****	
GAP	**0.172**	**0.154**	**0.111**	**0.164**	**0.065**	****

Bold numbers indicate statistical significance after sequential Bonferroni corrections

### Signatures of rapid local adaptation

**Fig. 2:**
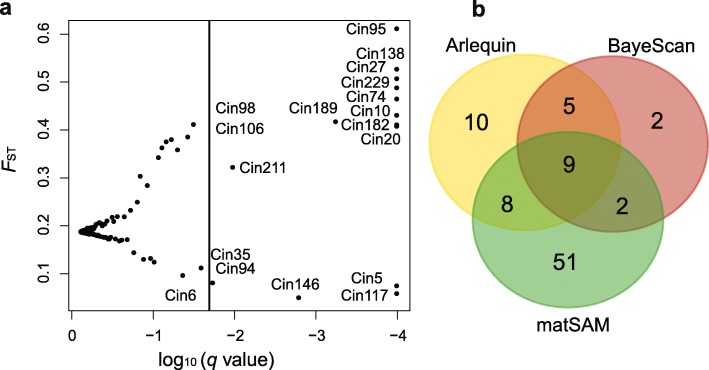
Microsatellite loci under selection. a, *F*_ST_-based outliers detected by BAYESCAN, and the solid vertical line represents false discovery rate of 0.05; b, the number of microsatellite loci under selection identified by two approaches (ARLEQUIN and BAYESCAN), as well as the environmental association analysis (matSAM)

Here, we used two genome scan approaches to detect the footprints of selection. Firstly, we identified a total of 32 outlier loci (21.9%) in ARLEQUIN, including 17 loci (11.6%) for directional selection and 15 loci (10.3%) for balancing selection (Additional file 1: Figure S3). Secondly, 18 outlier loci (12.3%) were detected based on the BAYESCAN approach, with 12 loci (8.2%) under directional selection and six loci (4.1%) under balancing selection (Fig. 2a). Interestingly, five out of nine identified outlier loci in BAYESCAN were located on the chromosome 1, suggesting a candidate genomic region under selection (Additional file 1: Figure S4). In total, 14 (9.6%) loci were commonly identified as *F*_ST_ outliers by both approaches (Fig. 2b).

**Fig. 3:**
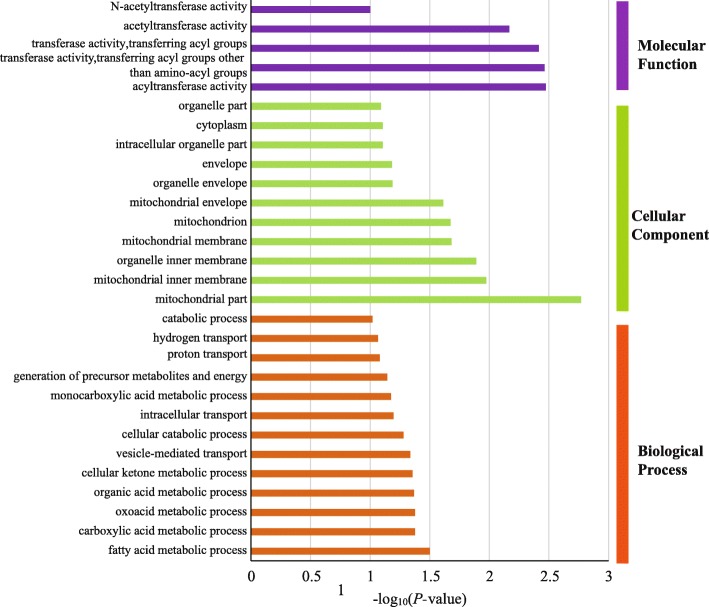
Gene ontology (GO) term enrichment analysis. The GO annotation results were based on 44 genes annotated by 19 adaptive outlier loci and within 20 kb selective sweep windows. Gene ontology categories included molecular function, cellular component and biological process. GO categories for each function were sorted by decreasing order of evidence, based on the GO enrichment test *P*-value

**Fig. 4:**
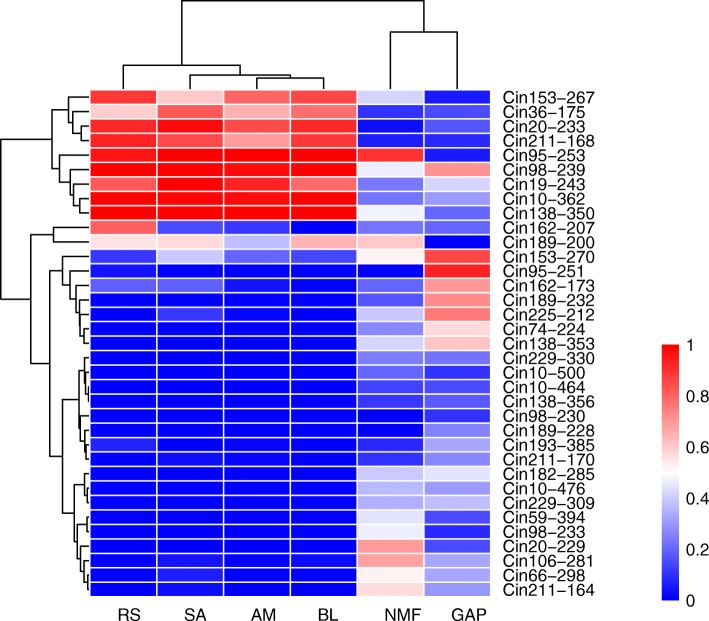
Heat map of allele frequencies of 35 alleles obtained from 19 adaptive loci. Rows represent specific alleles, and columns represent different populations. Colours represent normalized allele frequencies

**Fig. 5:**
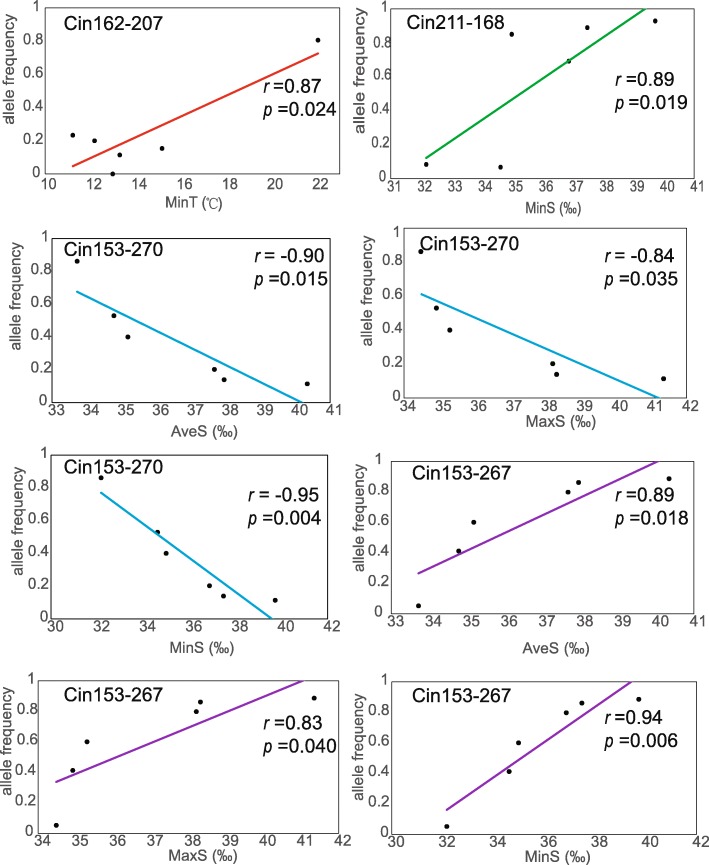
Pearson correlation tests between allele frequencies at adaptive loci and environmental factors. MinT, AveS, MaxS and MinS refer to the lowest monthly average water temperature, annual average water salinity, the highest monthly average salinity, the lowest monthly average salinity, respectively

In order to further assess environmental influence on outlier loci, spatial analysis method (matSAM) was performed to test the correlation between allelic frequency and environmental factors. Interestingly, a total of 93 alleles (9.5%) at 70 microsatellite loci (47.9%) showed significant association with at least one of the six environmental parameters (*P* < 0.05), indicating strong effects of local environmental factors on genetic variation. A total of 41 loci were associated with temperature, including 22, 6 and 33 loci for annual average water temperature (AveT), the highest monthly average water temperature (MaxT) and the lowest monthly average water temperature (MinT), respectively. A total of 57 microsatellite loci were detected with the strong correlation with salinity, including 40, 30 and 56 loci for annual average water salinity (AveS), the highest monthly average water salinity (MaxS) and the lowest monthly average water salinity (MinS), respectively. Altogether, 28 loci showed significant associations with both temperature and salinity. Furthermore, a total of 19 loci detected by ARLEQUIN or BAYESCAN approach were significantly associated with environmental factors (Fig. 2b). We used these 19 loci as adaptive outlier loci for further analyses because they were the best candidates associated with adaptive polymorphisms at neighboring genes potentially under environmental selection. Of these 19 adaptive outliers and within their selective sweep windows, a total of 44 genes were identified (Table 3; Additional file 1: Table S1). After GO term enrichment analysis, 29 GO terms were significantly enriched, such as metabolic process, ion transport, and catalytic activity (Fig. 3). In addition, four KEGG pathways were detected, including adherens junction, cell cycle, ubiquitin mediated proteolysis and Wnt signaling pathway. Across all 19 adaptive outlier loci, we detected allele frequency changes at a total of 35 alleles (Fig. 4). The allele frequency of several adaptive loci in the Red Sea population was changed (Fig. 4). For example, the frequency of allele 207 at the locus Cin162 (frequency = 0.81) was much higher in the Red Sea population than that in the other five populations (frequency = 0–0.23). A similar pattern was observed for the allele 270 at the locus Cin153 (frequency = 0.11) in the Red Sea population, which was much lower than that that in the others (frequency = 0.14–0.86). Interestingly, we detected significantly positive or negative correlations between allele frequency and environmental factors at the adaptive loci. For example, at the locus Cin162, the frequency of allele 207 (Cin162–207) was positively correlated with the minimum water temperature (Pearson’s *r* = 0.87, *p* = 0.024; Fig. 5). At the locus Cin211, the frequency of allele 168 (Cin211–168) had a positive correlation with the minimum salinity (Pearson’s *r* = 0.89, *p* = 0.019; Fig. 5). At the locus Cin153, the frequency of allele 270 (Cin153–270) was negatively correlated with salinity, including AveS (Pearson’s *r* = − 0.90, *p* = 0.015), MaxS (Pearson’s *r* = − 0.84, *p* = 0.035) and MinS (Pearson’s *r* = − 0.95, *p* = 0.004; Fig. 5). These patterns suggest that local environmental factors, such as temperature and salinity, could largely influence the allele frequency of adaptive loci, thus contributing to rapid microevolution.

**Table 3:** Gene annotation of 19 adaptive outlier loci against the *Ciona robusta* genome

Loci	Location	*E*-value	Gene annotation (NCBI BLASTN)	Uniprot GO/AmiGo2 GO
Cin10	Chromosome 1: 4,135,583-4,136,208	0	no hit	no
Cin19	Chromosome 1: 6,677,358-6,678,040	0	uncharacterized LOC108949898	no
Cin20	Scaffold HT001144.1: 27,547-27,960	0	protein MB21D2 (MB21D2 gene)	protein-containing complex binding; cadherin binding
Cin36	Scaffold HT000030.1: 142,799-143,299	0	no hit	no
Cin59	Scaffold HT000127.1: 84,997-85,135	1.00E-53	uncharacterized LOC104266650	no
Cin66	Chromosome 3: 2,342,118-2,342,780	0	uncharacterized LOC104265511	no
**Cin74**	**Chromosome 3:** **5,106,446-5,106,970**	**0**	**polyadenylate-binding protein 2 (PABP2 gene)**	**RNA binding; mRNA polyadenylation;** **poly(A) binding; nucleus; cytoplasm**
**Cin95**	**Chromosome 5:** **3,787,692-3,787,983**	**6.00E-122**	**S-phase kinase-associated protein 2 (SKP2 gene)**	**G1/S transition of mitotic cell cycle;** **G2/M transition of mitotic cell cycle;** **protein polyubiquitination; protein binding** **ubiquitin-protein transferase activity**
**Cin98**	**Chromosome 5:** **4,569,724-4,570,072**	**3.00E-170**	**low-density lipoprotein receptor-related protein 6 (LRP6 gene)**	**negative regulation of protein phosphorylation;** **low-density lipoprotein particle receptor activity;** **signaling receptor binding; frizzled binding; protein binding**
Cin106	Chromosome 6: 1,498,836-1,499,402	0	inositol polyphosphate 5-phosphatase OCRL-1 (OCRL gene)	GTPase activator activity; in utero embryonic development; photoreceptor outer segment; phosphatidylinositol-4,5-bisphosphate 5-phosphatase activity; trans-Golgi network; signal transduction; nucleus; clathrin-coated vesicle
Cin138	Chromosome 1: 1,567,428-1,567,684	6.00E-88	no hit	no
Cin153	Scaffold HT000103.1: 128,876-129,440	0	IST1 homolog (IST1 gene)	protein binding
**Cin162**	**Chromosome 9:** **4,578,790-4,579,144**	**2.00E-152**	**mitochondrial uncoupling protein 3 (UCP3 gene)**	**response to cold; adaptive thermogenesis; response to nutrient;** **lipid metabolic process; fatty acid metabolic process; aging;** **response to superoxide; response to hypoxia; respiratory gaseous exchange;** **oxidative phosphorylation uncouple activity; mitochondrial inner;** **transporter activity membrane**
Cin182	Chromosome 12: 1,101,323-1,101,594	2.00E-94	no hit	no
Cin189	Chromosome 13: 1,255,212-1,255,526	6.00E-144	no hit	no
**Cin193**	**Chromosome 14:** **244,529-244,945**	**0**	**histone acetyltransferase p300 (EP300 gene)**	**negative regulation of transcription by RNA polymerase II;** **response to hypoxia; somitogenesis;** **stimulatory C-type lectin receptor signaling pathway;** **RNA polymerase II activating transcription factor binding;** **RNA polymerase II proximal promoter sequence-specific DNA binding;** **p53 binding; histone acetyltransferase complex**
**Cin211**	**Chromosome 5:** **2,499,390-2,499,700**	**8.00E-126**	**AP-3 complex subunit mu-1** **(AP3M1 gene)**	**anterograde axonal transport; anterograde synaptic vesicle transport**
Cin225	Chromosome 10: 293,070-293,590	0	persulfide dioxygenase ETHE1 (ETHE1 gene)	glutathione metabolic process; hydrogen sulfide metabolic process; oxidation-reduction process; sulfur dioxygenase activity; iron ion binding; mitochondrion; nucleoplasm
Cin229	Scaffold HT000276.1: 9605-10,133	0	no hit	No

Gene annotation was performed using BLASTN search in the NCBI website and gene ontology (GO) terms were against UniProt database and AmiGO 2 GO browser. Microsatellite loci in bold are located in genes putatively involved in temperature and salinity adaptation

Most of these adaptive loci were annotated to genes involved in temperature and salinity adaptation (Table 3; Additional file 1: Table S1). For the adaptive locus Cin162, it was annotated as the mitochondrial uncoupling protein 3 gene (UCP3) located on the Chromosome 9 in *C. robusta*. This gene is involved in metabolic process and especially adaptive thermogenesis in response to cold stress based on GO annotation according to Uniprot or AmiGo2 database (Table 3). The S-phase kinase-associated protein 2 gene (SKP2), which was the best gene hit for the locus Cin95, plays a key role in mitotic cell cycle and protein ubiquitination. Another locus Cin211 was located within the region of the AP-3 complex subunit mu-1gene (AP3M1), and the AP3M1 gene, which regulates the process of protein metabolism and Rab GTPase binding, is involved in salinity stress tolerance. In addition, two genes were detected in the up-stream of the locus Cin74 (Additional file 1: Table S1): one gene was ubiquitin-conjugating enzyme E2C gene (UBE2C), which was reported to get involved in ubiquitin-dependent protein catabolic process under heat stress in *Ciona* ascidian, and another one was the telomere length regulation protein TEL2 gene (TELO2) with the functions of DNA damage response and Hsp90 protein binding. Indeed, many other genes play crucial roles in various important biological processes related to responses to environmental stresses, such as cell apoptosis, fatty acid metabolic process, and ion transport (Table 3; Fig. 3; Additional file 1: Table S1). Altogether, we detected functional genes associated with these adaptive loci that were involved in the process of rapid adaptation to the harsh environmental conditions in the Red Sea.

## Discussion

Deep insights into the rapid adaptive changes during range expansion will facilitate our understanding on species’ distribution ranges and responses to future environmental changes [3, 15, 18, 45]. Based on the genome-wide gene-associated microsatellites, we investigated genetic signatures of rapid adaptation to harsh environments of the Red Sea in *C. robusta*. The Red Sea population was significantly differentiated from the other populations (Table 2; Fig. 1), and the genome-wide scan using multiple methods identified a set of adaptive outlier loci (Fig. 2; Additional file 1: Figure S3). Interestingly, the allele frequency at several adaptive loci in the Red Sea population was largely altered (Fig. 4), and we found significant correlations between allele frequency and local environmental factors at these adaptive loci (Fig. 5). Furthermore, many genes were annotated to get involved in local temperature and salinity adaptation, and biological processes and pathways of adaptive genes may underline the effect of rapid adaptive evolution (Table 3 and Additional file 1: Table S1; Fig. 4). All the evidence obtained in this study clearly showed that environment-driven selection had left detectable signatures in the genome of *C. robusta* within a few generations, thus supporting the hypothesis that local environmental adaptation can rapidly occur, and such a rapid microevolutionary process may largely contribute to invasion success in aquatic ecosystems (i.e., rapid microevolution hypothesis).

### Factors for population genetic structure in the Red Sea

For newly established populations, gene flow and some events such as genetic bottleneck, genotype sorting and drift are likely to influence population genetic structure [46–48]. Given the increasing shipping activities, especially through the Suez Canal, human-mediated dispersal could facilitate gene flow between populations of the Red Sea and Mediterranean Sea [16, 41, 49]. According to Shenkar et al. [35], mtDNA analyses of *C. robusta* samples from the same sampling site in 2015 suggested that the Red Sea population might be introduced from the Mediterranean Sea, where *C. robusta* were detected much earlier in the end of nineteenth century [31, 44]. In our study, the Red Sea population showed significant genetic divergence with the two populations from the Mediterranean Sea (Table 2; Fig. 1). Such a pattern suggests that gene flow might not occur, or local adaptation has largely overridden the effect of gene flow as we detected strong influence of local environmental factors on both loci and associated allele frequency (Figs. 2, 4 and 5). For novel populations with a small number of colonists, they may experience a dramatic decline in genetic diversity due to bottleneck effects, which can contribute to population genetic divergence by retaining a non-random set of genotypes when compared to their original populations [50]. Although we detected reduced population genetic diversity in the Red Sea population when compared with two Mediterranean populations (AM, BL), further analyses showed no signal of recent bottleneck in the Red Sea population, indicating that genetic bottleneck might not be the major factors for the observed genetic divergence of the Red Sea population. Although we could not exclude the potential influence of other processes, such as genotype sorting and genetic drift, multiple lines of evidence in this study clearly supported that natural selection caused by harsh environments had largely influenced the population genetic patterns. Strong selection pressures by environmental challenges, such as global warming, marine pollution and ocean acidification, can result in high population genetic divergence for marine species [4, 51, 52]. Here, we detected significant environmental difference between the Red Sea population and the others. The Red Sea population was firstly detected in 2015 and has experienced ~ 10 generations after introduction. We identified a large number of adaptive loci which exhibited significant correlation with water temperature and salinity, indicating a strong environment-driven selection in the genome of *C. robusta* in a short timescale. Similar strong and rapid adaptation was also detected in other marine species. For example, significant reproductive isolation was detected within 13 generations among introduced salmon populations in novel environments [53]. Bernardi et al. [16] reported that rapid adaptive evolution promoted the bluespotted cornetfish to rapidly invade over the Mediterranean Sea in less than 7 years, and they further revealed adaptive genomic regions under salinity selection, such as osmoregulatory regions. Results obtained in this study, as well as evidence from related investigations, suggest that rapid local adaptation could rapidly occur within several generations when selection pressures are high.

### Sources of genetic variation in rapid environment-driven adaptation

Rapid local adaptation during biological invasions primarily depends on two sources of genetic variation: standing genetic variation and new beneficial mutations [7, 13, 14, 54]. A large number of studies suggested that rapid local adaptation induced by recent natural selection and/or strong selection pressure predominantly relied on standing genetic variation, by which the beneficial alleles were immediately available at higher frequencies compared to new mutations [10, 55]. For the Red Sea population, standing genetic variation could provide preadapted genotypes, such as the genotypes well-suited to high temperature and salinity, to rapidly adapt to the harsh environment in the Red Sea. In addition, Lin et al. [44] revealed that the length of selective sweeps on the genomic regions in *C. robusta* could be as narrow as 9.8 kb, suggesting the soft selective sweep with standing genetic variation. However, recent studies documented that novel mutations could provide opportunities, especially for small founding populations, to rapidly adapt to the new environments [56]. *C. robusta* exhibits a high level of mutation rate, accompanied with relatively high fecundity, rapid growth rate and short life history, which may provide new mutations for natural selection [26, 57]. Thus, we could not rule out the effects of new mutations in environment-driven selection, and more than one type of genetic variation might get involved in the rapid local adaptation in the Red Sea population.

### Candidate genes for rapid local adaptation

Understanding the function of adaptive genes can provide insights into the genetic basis of rapid microevolution in changing environments [12, 44]. In our study, many candidate genes were identified with important roles in rapid adaptation to temperature and salinity (Table 3 and Additional file 1: Table S1). For example, one strong candidate locus (Cin162) was annotated to mitochondrial uncoupling protein 3 gene (UCP3), and this gene is especially involved in cold stress response. UCP3-mediated effects on adaptive thermogenesis under cold stress have been discovered in marine fish, mainly related to fat metabolism [58, 59]. Interestingly, the allele frequency of this locus (Cin162–207) was detected to be significantly correlated with the minimum temperature (MinT) with much higher allele frequency in Red Sea population than that in the others. This pattern supported that local environments could largely alter allele frequency of functional genes, thus contributing to the rapid microevolution. Another two important genes involved in high temperature adaptation, ubiquitin-conjugating enzyme E2C gene (UBE2C) and telomere length regulation protein TEL2 gene (TELO2), were detected within the selective sweep window of the locus Cin74. For the UBE2C gene, it encodes the protein that mainly plays a role in ubiquitin-dependent protein catabolic process [60, 61]. According to Lopez et al. [61], the ubiquitin-mediated proteolysis pathway, including ubiquitin-conjugating enzyme E2, was enriched by the proteomic analyses of *C. intestinalis* in high temperature treatment. In addition, this gene can cooperate with Cullin-type ubiquitin ligase to get involved in spermatogenesis and fertilization in *C. intestinalis* [60, 62]. Another gene TELO2, which can participate in the cellular resistance to DNA damage, especially interactions with the Hsp90 protein, is likely to play a crucial role in coping with thermal stress in the Red Sea population. In addition, we detected a large number of genes involved in salinity adaption. For example, the AP-3 complex subunit mu-1gene (AP3M1) with a much higher allele frequency of the locus Cin211 in the Red Sea population, encodes the AP-3 protein, which is linked to the Golgi region as an adaptor-related protein complex. The ion transport between the *trans*-Golgi network and the plasma membrane was required for salinity stress adaptation [63, 64]. This gene is also connected to the RABA1 GTPase region, which can get involved in the regulation of ion homeostasis in Arabidopsis [63, 64]. Two genes, histone acetyltransferase p300 gene (EP300) and low-density lipoprotein receptor-related protein 6 gene (LRP6), were categorized into the Wnt signaling pathway. The Wnt signaling pathway in *C. intestinalis* includes at least three pathways: the canonical pathway, the planar cell polarity (PCP) pathway and the Wnt/Ca^2+^ pathway based on the KEGG database (the Kyoto Encyclopedia of Genes and Genomes, KEGG). Multiple studies have revealed that the Wnt signaling pathway played a crucial role in many biological processes, such as cell proliferation [65], embryogenesis [66], and biomineralization [67]. A recent proteomic analysis of the Pacific white shrimp suggested that the Wnt pathway could participate in low salinity adaptation [68]. Thus, further transcriptomic and proteomic investigations on *C. robusta* under high temperature and salinity in laboratory conditions can contribute to our understanding which adaptive genes and related biological processes and pathways play a key role in rapid microevolution.

## Conclusions

Although *C. robusta* is generally considered as a temperate-water species, it has currently successfully established in a Red Sea marina since 2015. Rapid local adaptation is a crucial mechanism for invasive species to cope with novel environments and/or environmental stresses during range expansion. Although it is well-known that phenotypic plasticity is a crucial mechanism for temporal adjustment during range expansion, while microevolution is more important for successful population establishment in a long-term perspective. Interestingly, the results obtained in our study suggest that rapid microevolution could occur within a few generations. We detected a large number of adaptive loci and candidate genes responsible for temperature and salinity adaptation in *C. robusta*, and these genes are candidate loci for further studying adaptation dynamics based on single genes or gene networks using *C. robusta* as a model. Our study confirms the rapid microevolution hypothesis that rapid local adaptation is largely responsible for the harsh environmental adaptation and therefore contributes to invasion success in different aquatic ecosystems with largely varied environmental factors.

## Materials and methods

### Sampling and species identification

*C. robusta* samples were collected from the Eilat marina (29°33′11.3″ N, 34°57′35.9″ E) on March 2018 (experiencing ~ 10 generations after introduction). All collected individuals were immediately preserved in absolute ethanol at 4 °C for further genetic analyses. Total genomic DNA was extracted using a DNeasy Blood and Tissue Kit (Qiagen). To confirm the species identification based on morphology, one mtDNA fragment cytochrome *c* oxidase subunit 3–NADH dehydrogenase subunit 1 (COX3-ND1) [33, 69], was used to perform molecular identification. The COX3-ND1 segment was amplified by the primers TX3F and TN1R following the protocol as described in Zhan et al. [33].

### Genome-wide gene-associated microsatellites genotyping

Here, we used a total of 146 genome-wide gene-associated microsatellite makers [44, 70] to genotype the Red Sea population. Specially, six microsatellite markers (Cin54, 76, 92, 125, 210, 213) were excluded from the Lin et al. [44] dataset because of poor PCR amplifications for the Red Sea individuals. PCR amplification and microsatellites genotyping were performed based on the protocol as described in Lin et al. [44, 70]. Amplified fragments were separated on an ABI 3730xl automated sequencer with the GeneScan™ 500 LIZ™ internal size standard. Alleles were scored with GeneMapper® software v.4.0 (Applied Biosystems). For the other five populations (two Mediterranean populations: AM, BL; one Atlantic population: SA; two Pacific population: NMF, GAP; Table 1), the datasets were adopted from our previous study [44].

Water temperature and salinity are among the most crucial environmental variables in marine ecosystems, as these factors largely influence survival, development and many physiological processes of marine species. The Red Sea was characterized with a high level of temperature and salinity [35]. Therefore, the average values of six environmental factors (among 1955–2012) associated with sea surface temperature (SST) and salinity were obtained from the National Oceanic and Atmospheric Administration (NOAA; http://www.nodc.noaa.gov/OC5/SELECT/woaselect/woaselect.html), including three metrics of SST: annual average water temperature (AveT), the highest monthly average water temperature (MaxT), the lowest monthly average water temperature (MinT) and three metrics of salinity: annual average water salinity (AveS), the highest monthly average water salinity (MaxS), the lowest monthly average water salinity (MinS). We used a non-parametric Kruskal-Wallis test to assess differences in temperature and salinity between the Red Sea population and the others in SPSS v.18. In addition, a principal component analysis (PCA) with the ‘prcomp’ package in program R was performed to illustrate the high salinity and temperature in Red Sea population.

### Population genetic patterns

Population genetic diversity was measured by allelic richness (*A*_R_), observed heterozygosity (*H*_O_), expected heterozygosity (*H*_E_) and inbreeding coefficient (*F*_IS_) using FSTAT v. 2.9.3.2 [71]. The non-parametric Kruskal-Wallis test was used to test the difference in allelic richness (*A*_R_) between the Red Sea population and the others. Recent bottleneck effect was tested using program Bottleneck v.1.2.02 [72] based on the two-phase model (TPM) with 90% one-step mutations, and statistical significance was based on 1000 iterations with a one-tailed Wilcoxon test. In addition, we performed the mode shift test, and deviation from L-shaped distributions of allele frequency would suggest recent bottlenecks using Bottleneck program [73].

We assessed population genetic differentiation with pairwise *F*_ST_ values using 10,000 permutations in ARLEQUIN and the significance level was adjusted after sequential Bonferroni correction. To further investigate population genetic structure, we performed the Bayesian clustering in STRUCTURE v.2.3 [74]. For the STRUCTURE analysis, we assessed likelihoods for models with the number of clusters ranging from *K* = 1 to 6 (the total number of populations), with 100,000 Markov chain Monte Carlo iterations preceded by 100,000 burn-in, and we performed ten independent runs for each *K* value. In addition, we further conducted Bayesian analyses to assess population assignment between the Red Sea population and Mediterranean-Atlantic population group. The optimal *K* value was identified with STRUCTURE HARVESTER [75, 76]. The program DISTRUCT [77] was used to visualize the results.

### Identification of candidate adaptive loci under selection

In this study, two different population differentiation approaches were used to search for microsatellite loci putatively under selection. Firstly, we screened all microsatellite loci with the fdist2 method under a hierarchal island model in ARLEQUIN [78]. This analysis was simulated based on 1000 demes with 50,000 simulations. Secondly, we identified outlier loci based on a Bayesian approach in BAYESCAN v.2.1 [79] according to default parameter settings. Candidate loci under selection were defined as those with false discovery rate lower than 5% (*q*-value < 0.05).

In order to identify loci that have strong association with particular environmental variables and further confirm that the outlier loci were the outcome of selection resulting from local environment factors, the environmental association analysis (EAA) was conducted in MatSAM v.2 [80]. MatSAM uses multiple univariate logistic regressions to test the associations between the allelic frequency and environmental variable at particular loci with the Bonferroni correction at a confidence level of 95% based on the cumulated test. Pearson correlations were performed in SPSS v.18 to test the correlation between the allele frequency and environmental factor at the adaptive loci.

### Adaptive outlier loci annotation

To annotate gene functions of the putatively adaptive outlier loci, we firstly located these loci on the chromosomes based on the *C. robusta* KH assembly of Satou et al. [81] at Ensembl (www.ensembl.org). In addition, we chose 20 kb up- and down-stream of each adaptive outlier loci as a selective sweep window against the *C. robusta* KH assembly as suggested by Lin et al. [44], and then we obtained the sequences of the selective sweep windows at Ensembl. Furthermore, sequences of candidate microsatellite loci and selective sweep windows were subjected for BLASTN search at the NCBI website to obtain the best gene hits. Finally, UniProt database and AmiGO 2 GO browser [82, 83] were used to conduct gene annotation and then assign gene function based on gene ontology (GO) terms. We performed GO enrichment and KEGG pathways analysis using DAVID 6.7 [84].

## Additional files

Additional file 1: **Figure S1.** (a) Temperature and (b) salinity data in six populations. **Figure S2.** PCA plot on environmental factors for six *Ciona robusta* populations. **Figure S3.** Outlier detection in 146 microsatellite loci in ARLEQUIN. Purple line represents 99% confidence intervals; red and green lines represent 95 and 5% confidence intervals, respectively. **Figure S4.** Manhattan plot showing the distribution of *F*_ST_-based outliers detected by BAYSECAN across different chromosomes of *Ciona robusta* KH assembly. The *q*-value of given locus is the minimum false discovery rate (FDR) at which this locus may become significant. **Table S1.** Protein MB21D2 (MB21D2 gene). (ZIP 1091 kb)
