# Supplementary figures and images for: Rapid microevolution during recent range expansion to harsh environments
Source: BMC Evol Biol. 2018 Dec 7;18:187. doi: 10.1186/s12862-018-1311-1 (PMC6286502; doi:10.1186/s12862-018-1311-1)

**a**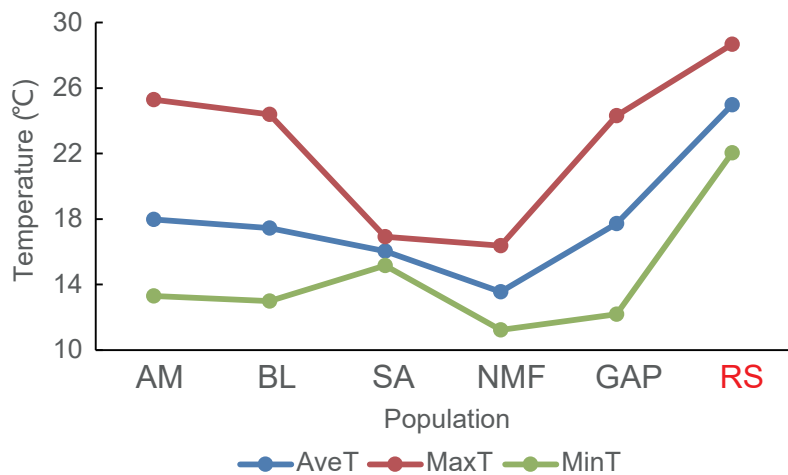**b**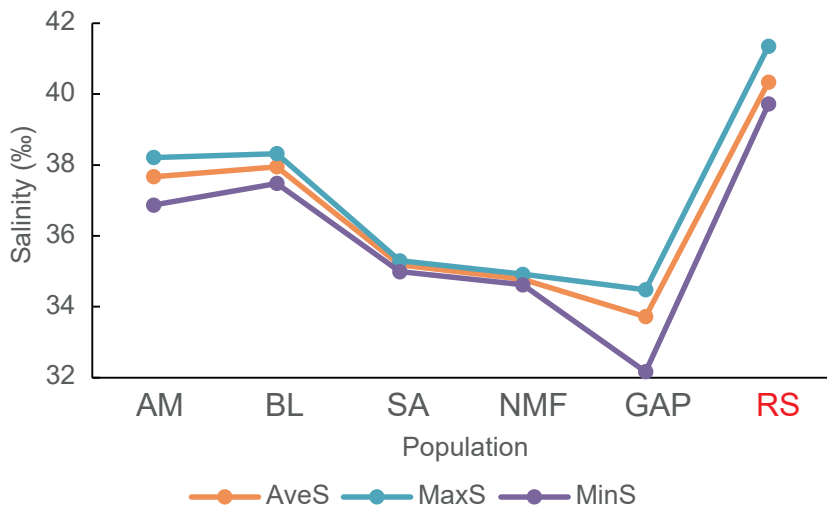

Supplement: Supplementary file 1 — Figure S1. (a) Temperature and (b) salinity data in six populations. Figure S2. PCA plot on environmental factors for six Ciona robusta populations. Figure S3. Outlier detection in 146 microsatellite loci in ARLEQUIN. Purple line represents 99% confidence intervals; red and green lines represent 95 and 5% confidence intervals, respectively. Figure S4. Manhattan plot showing the distribution of FST-based outliers detected by BAYSECAN across different chromosomes of Ciona robusta KH assembly. The q-value of given locus is the minimum false discovery rate (FDR) at which this locus may become significant. Table S1. Protein MB21D2 (MB21D2 gene). (ZIP 1091 kb) [file 12862_2018_1311_MOESM1_ESM.zip › Fig. S1.pdf]

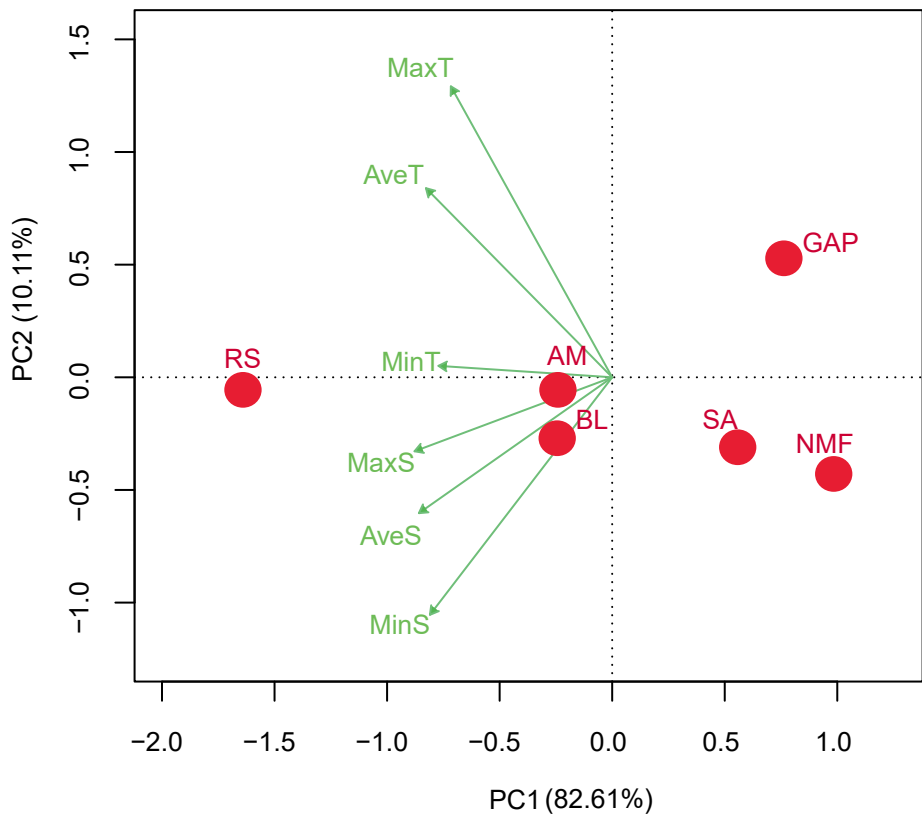

Supplement: Supplementary file 1 — Figure S1. (a) Temperature and (b) salinity data in six populations. Figure S2. PCA plot on environmental factors for six Ciona robusta populations. Figure S3. Outlier detection in 146 microsatellite loci in ARLEQUIN. Purple line represents 99% confidence intervals; red and green lines represent 95 and 5% confidence intervals, respectively. Figure S4. Manhattan plot showing the distribution of FST-based outliers detected by BAYSECAN across different chromosomes of Ciona robusta KH assembly. The q-value of given locus is the minimum false discovery rate (FDR) at which this locus may become significant. Table S1. Protein MB21D2 (MB21D2 gene). (ZIP 1091 kb) [file 12862_2018_1311_MOESM1_ESM.zip › Fig. S2.pdf]

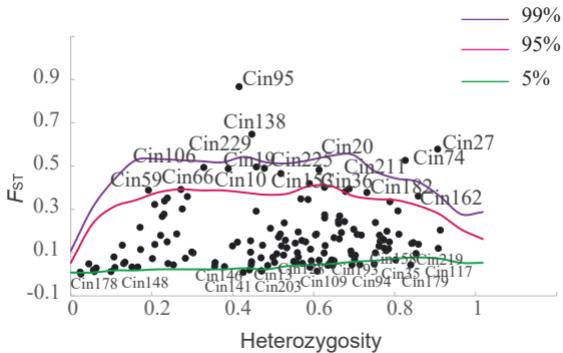

Supplement: Supplementary file 1 — Figure S1. (a) Temperature and (b) salinity data in six populations. Figure S2. PCA plot on environmental factors for six Ciona robusta populations. Figure S3. Outlier detection in 146 microsatellite loci in ARLEQUIN. Purple line represents 99% confidence intervals; red and green lines represent 95 and 5% confidence intervals, respectively. Figure S4. Manhattan plot showing the distribution of FST-based outliers detected by BAYSECAN across different chromosomes of Ciona robusta KH assembly. The q-value of given locus is the minimum false discovery rate (FDR) at which this locus may become significant. Table S1. Protein MB21D2 (MB21D2 gene). (ZIP 1091 kb) [file 12862_2018_1311_MOESM1_ESM.zip › Fig. S3.pdf]

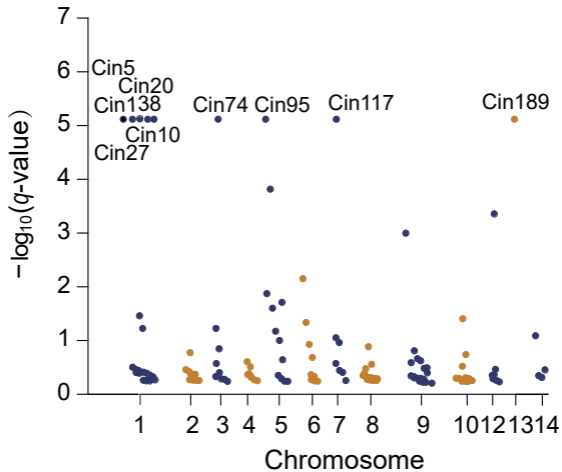

Supplement: Supplementary file 1 — Figure S1. (a) Temperature and (b) salinity data in six populations. Figure S2. PCA plot on environmental factors for six Ciona robusta populations. Figure S3. Outlier detection in 146 microsatellite loci in ARLEQUIN. Purple line represents 99% confidence intervals; red and green lines represent 95 and 5% confidence intervals, respectively. Figure S4. Manhattan plot showing the distribution of FST-based outliers detected by BAYSECAN across different chromosomes of Ciona robusta KH assembly. The q-value of given locus is the minimum false discovery rate (FDR) at which this locus may become significant. Table S1. Protein MB21D2 (MB21D2 gene). (ZIP 1091 kb) [file 12862_2018_1311_MOESM1_ESM.zip › Fig. S4.pdf]
